# Supplementary material for: Individual differences in trait creativity moderate the state-level mood-creativity relationship
Source: PLoS One. 2020 Aug 3;15(8):e0236987. doi: 10.1371/journal.pone.0236987 (PMC7398526; doi:10.1371/journal.pone.0236987)
Supplement: Attachment — Submitted filename: Response to Reviewers.docx [file pone.0236987.s002.docx]

Dear Dr. Carlos Andres Trujillo,

Enclosed please find our revised manuscript (PONE-D-19-31431) entitled “Individual Differences in Trait Creativity Moderate the State-Level Mood-Creativity Relationship " for publication in PLoS ONE.

First of all, we would like to thank you, and the two reviewers for the time and effort spent in reviewing our manuscript. Your insightful comments have helped us to significantly improve the manuscript.

Please find a point by point reply below. We think that we have been able to address all comments and hope that our manuscript is now ready for publication in PLoS ONE.

Sincerely yours on behalf of all authors,

Dan Zhang, Ph.D.

Associate Professor,

Department of Psychology,

Tsinghua University, Beijing, 100084, China

Tel: +86-10-62796737

E-mail: dzhang@tsinghua.edu.cn

**Comments by the academic editor:**

You will find very useful comments from the reviewers from which I want to highlight 1) the need to explain your work in contrast to the literature mentioned by reviewer 1, and 2) explain very clearly your rationale for choosing multilevel analysis instead of panel data as requested by reviewer 2. I would really appreciate a special effort to explain your findings in a much clearer way. Following the tables is very hard.

Reply: The comments raised by the two reviewers are replied below. Regarding your suggestion on the presentation of the results, we have added a new figure (Fig. 3) to visualize the moderation model from the Table.

**Comments by Reviewer #1:**

The manuscript needs one more careful reading to address some typographical and grammatical errors.

Reply: We have used the Grammarly service to check the manuscript and invited an English expert to proofread our revised manuscript.

Further, it needs to give additional acknowledgement to the extensive work done by Isen and associates on the relationship between mood and creativity, much of which was subsumed in the Bass et al. meta analysis, and to comment on how the findings align with findings from DeDreu et al (2008) when it comes to how different emotional states can influence creative outputs through different channels. A more generous discussion on how findings from this work align with earlier work will affirm the current works contribution to this important research area.

Reply: The classical work by Isen and associates is now included in Introduction (line 40-48) and we have discussed the present result under the framework of the dual pathway model proposed by DeDreu (line 516-523). In addition, the last part of Introduction was re-organized into a hypothesis-driven way and the Discussion section was revised to more explicitly highlight possible contribution of the present study.

Again, many thanks for your constructive comments!

**Comments by Reviewer #2**

In the study “individual Differences in Trait Creativity Modulate the State-Level Mood-Creativity Relationships”, the authors state that creativity, far of being a stable state, depends on contextual factors. The authors focus on mood, one factor that enhances creativity with differences according its dimensions of study (). The authors suggest that a complete understanding of this relationship should consider trait-level factors, whose relevance has been widely demonstrated in other contexts. Thus, the authors empirically explore the influence of creativity traits on the relationship state mood. The findings of the study support that creativity trait has a moderator effect differential according to the creativity state-level.

In the introduction the authors justify the importance of the study for practice. The introduction of state level creativity and mood is concrete and very well explained. Although from the introduction the importance of creativity trait is set, with the strong emphasis on the exploratory character of the study the authors miss the opportunity to make a theoretical construction. This empirical emphasis privates the authors of exploring why to understand how creativity trait from its meaning may be relevant for the academic community.

Reply: The last part of Introduction has been revised to propose a hypothesis about the specific moderation effects (line 113-118).

This theoretical construction is not developed in the document. The authors should note that academics are committed to the generation of knowledge, which goes beyond the focus on practical implications of trait-level factors from specific domains. (p.4).

Reply: We made the following efforts to address the potential theoretical contributions of the present study:

1) The 2nd and 3rd paragraphs of Introduction have been slightly adjusted to be more focused on reviewing the theoretical explanations of mood-creativity relationship (line 40-48, line 82-84).

2) The Discussion section has been re-organized to first comment on the mood-creativity correlation, then to discuss the individual difference, and finally to integrate the results under the framework of the dual pathway to creativity model.

A minor general comment looks at to standardize the used terms. For example, modulate used as synonym of moderate. If modulation is different to moderation, it is necessary to explain why. The use of modulate distracts, because moderation is the most common term in academic community. It is possible that the authors are talking about interaction, which is slightly different from moderation. However, introducing a methodological term with scarce use is confusing.

Reply: All changed to moderate as suggested.

Regarding the methodology, the authors have an interesting sample of repeated measured to test the exploratory models. It is necessary to understand if the authors foresee any bias in the fact that the sample is composed of psychology students who use to be involved with the variables of the study.

Reply: A paragraph on limitation has been added in Discussion. The sample bias issue is mentioned together with some other issues that are worthy to be addressed in future studies.

Given the authors’ interest in the impact of creativity trait as moderator of state mood creativity relationship and that they have one measure of creativity trait for each of the 56 participants (repeated measures), they use multilevel analysis to test these interactions. There are some elements that deserve consideration by the authors in this regard. In multilevel methods observations are nested in contexts that influence differently their behavior. An important outcome of multilevel methods is to identify the environmental variables (2nd level) that influence individual results (1st level). In this study the observations are nested because they belong to the same individual. Although this procedure is statistically appropriate, the authors should consider explaining the bias originated in that the second-level variable (creativity trait), of course, emerges from the same individual who also reported mood and creativity-state during multiple stages of the research. In other words, there are not independence between dependent and independent variables. Furthermore, the independent variable is common for several observations.

Reply: We conducted an additional analysis between trait-level and state-level creativity and the results suggested sufficient independence at these two levels (line 268-274):

“Table 2 shows the participant-wise correlations between trait-level and state-level creativity. The average and variance of the state-level creativity ratings (originality and usefulness) per participant were calculated and then correlated with the corresponding trait-level creativity (RAT and AUT scores). Only weak and non-significant correlation coefficients were obtained, suggesting no systematic changes of one’s daily state rating patterns by his/her trait-level creativity. In other words, these correlation results demonstrated sufficient independence among these variables for the subsequent multi-level analysis.”

The results were summarized in a new Table 2 and added in the manuscript.

Second, given that this study is not interested in understanding how much variance is explained at each level, how and why multilevel method provides better outcomes than a panel with fix-effects by individuals?

Reply: The rationale for our selection mainly follows the tradition in previous psychological studies on individual difference. We made this clear in the revised manuscript (line 222-229):

“It should also be noted that the multi-level modeling method rather than the panel analysis method with fixed effects was employed to approach the problem of cross-level interaction. The multi-level modeling method is believed to be more suitable for consideration of the research assumption of the present study. Specifically, the mood-creativity links (β_1j_ and β_2j_) were explicitly expressed as a random effect, emphasizing a quantitative description of these links by the participants’ individual difference by their trait creativity. Such an assumption is in line with most previous studies on individual difference in the field of psychology (Chi et al., 2015; Conner & Silvia, 2015) and allows for a better generalizability of the findings beyond the sample population (Antonakis et al., 2019; Heisig et al., 2017; Snijders & Bosker, 2011).”

For the previous two points I strongly recommend the authors to review any edition of Snijders and Bosker’s book.
- Snijders, T. A., & Bosker, R. J. (2011). Multilevel analysis: An introduction to basic and advanced multilevel modeling. Sage.

Regarding the results it should be noted that according to the Table 1 there are strong correlations between the variables AUT_FLU, AUT_FLE and AUT_ORI and between RAPM and RAT. However, multi-collinearity is not mention in the manuscript and the author(s) should guarantee that there are not multicollinearity effects associated with the results.

Reply: We calculated the variance inflation factor (VIF) of the variables AUT_FLU, AUT_FLE, AUT_ORI, RAPM and RAT by using a simple regression model. The result is shown in the table below. As all VIF were less than the empirical threshold of 10. Considering the interpretability of the original variables, we keep them in the multi-level model (line 217-221):

“Note that the trait-level variables in the above models were the original trait creativity scores (and intelligence in the second model). Although significant pairwise correlations between some of these trait-level variables were observed (see Results, Table 1), the problem of multicollinearity was not severe, with the maximal variance inflation factor (VIF) being 7.5 (less than the empirical threshold of 10).”

**Table. VIF values of trait variables**

|  | RAPM | AUT_FLU | AUT_FLE | AUT_ORI | RAT |
| --- | --- | --- | --- | --- | --- |
| VIF | 1.30 | 7.52 | 1.47 | 6.79 | 1.28 |

Additionally, the authors should review the notation in Table 2. There are signs in the table without interpretation in the note. Additionally, the note is confusing. It indicates Model 1 is the multilevel model without covariate, and model 2 is the model using RAMP score as covariate. However, both models include covariable and the contrast between the two models is the inclusion of RAMP as an additional covariable.

Reply: The only difference between model 1 and model 2 is the inclusion of RAMP. The confusing note has been removed. The signs and their explanations have been checked and corrected.

Although the graphs the authors present seem to have interesting information confirming the significant interactions of the results, the decision to focus on Model 1 (from table 2) is not explained.

Reply: We agree it would be more proper to focus on Model 2 and the corresponding report in the main text has been updated (line 281-295). Accordingly, one of the results was not significant in Model 2 therefore removed from our results report. Nevertheless, the interpretation of our main findings was still valid.

The discussion section replicates the results section. Considering that the theoretical construction was not strong in the introduction, it is expected that construction to be tackled in the discussion section.

Reply: The Discussion section has been re-organized to emphasize possible theoretical contribution of the present findings.

Finally, What are the conclusions of your study?

Reply: A summary paragraph has been added at the end of Discussion (line 572-577).

In an attempt to summarize the previous comments, I suggest to strengthen the study by developing further theoretical construction from the exploratory results and to evaluate the appropriateness of the methodological procedures, highlighting the possible weak elements and showing how the authors dealt with them.

Reply: Many thanks for your constructive comments!
