# Supplementary material for: Individual differences in trait creativity moderate the state-level mood-creativity relationship
Source: PLoS One. 2020 Aug 3;15(8):e0236987. doi: 10.1371/journal.pone.0236987 (PMC7398526; doi:10.1371/journal.pone.0236987)
Supplement: Attachment — Submitted filename: Reply-Final.docx [file pone.0236987.s003.docx]

Dear Dr. Carlos Andres Trujillo,

Enclosed please find our revised manuscript (PONE-D-19-31431) entitled “Individual Differences in Trait Creativity Moderate the State-Level Mood-Creativity Relationship " for publication in PLoS ONE.

Again, we would like to thank you, and the reviewers for the time and effort spent in reviewing our manuscript. Your further comments regarding Table 1 and 2 have been properly addressed.

We think that we have been able to address all comments and hope that our manuscript is now ready for publication in PLoS ONE.

Sincerely yours on behalf of all authors,

Dan Zhang, Ph.D.

Associate Professor,

Department of Psychology,

Tsinghua University, Beijing, 100084, China

Tel: +86-10-62796737

E-mail: dzhang@tsinghua.edu.cn

**Comments by the academic editor:**

I just need you to revise tables 1 and 2 to improve clarity. In table 1 there are 6 correlations in the upper right side that provide inconsistent information (e.g. the correlation between S_use and S_ori present two different values). For table 2 please provide significance levels and the type of correlation calculated. Given the relatively low sample size, pearson correlation may not be appropriate, please clarify which type of correlation was used.

Reply:

The “inconsistent” information was due to the different calculation methods for the upper right and lower left part of the table. Different information was presented in the two parts, as explained in the main text (line 206-208): “Table 1 also shows the correlation at both between-participant (i.e. with each participant’s state-level data) and cross-sectional (i.e. with all participants’ momentary, state-level data pooled together) levels.”

In addition, we have improved the explanations in the footnote of Table 1: “Correlation coefficients below the diagonal are calculated at the between-participant level, in which the state-level variables (S_ori, S_use, PA, NA) were averaged within participants and the correlated with the trait-level variables across all participants (*N* = 54 for the correlation analyses). Cross-sectional correlations are presented above the diagonal, in which the state-level data were pooled together across participants (*N* =1317 for the correlation analyses). Pearson’s correlation was used.”

All the results in Table 2 were non-significant, as stated in the main text (line 222-223): “Only weak and non-significant correlation coefficients were obtained, suggesting no systematic changes of one’s daily state rating patterns by his/her trait-level creativity.” Here the sample size was 54 (the number of participants). Following your suggestion, we further calculated the Spearman’s correlation, and the results are shown below. All of these Spearman’s correlations are still weak and non-significant. As this piece of information only served as a control analysis and Pearson’s correlation was employed for the other correlational analyses in the present study, we prefer to keep the current results by Pearson’s correlation in the main text.

Table. Participant-wise correlations between trait-level and state-level creativity by Spearman’s correlation

|  | AUT_FLU | AUT_FLE | AUT_ORI | RAT |
| --- | --- | --- | --- | --- |
| M(S_ori) | 0.18 | -0.04 | 0.10 | 0.17 |
| M(S_use) | 0.06 | -0.02 | -0.08 | -0.11 |
| Var(S_ori) | -0.03 | 0.21 | -0.05 | -0.16 |
| Var(S_use) | -0.04 | 0.22 | -0.03 | -0.01 |
